# Simultaneously Intelligent Sensing and Beamforming Based on an Adaptive Information Metasurface

**DOI:** 10.1002/advs.202306181

**Published:** 2023-12-08

**Authors:** Rui Zhe Jiang, Qian Ma, Ze Gu, Jing Cheng Liang, Qiang Xiao, Qiang Cheng, Tie Jun Cui

**Affiliations:** ^1^ State Key Laboratory of Millimeter Waves Institute of Electromagnetic Space Southeast University Nanjing 210096 China; ^2^ Zhangjiang Laboratory 100 Haike Road, Pudong Shanghai 201210 China

**Keywords:** adaptive metasurface, information metasurface, intelligent sensing, intelligent wave manipulation, physical layer

## Abstract

Due to its ability to adapt to a variety of electromagnetic (EM) environments, the sensing‐enabled metasurface has garnered significant attention. However, large‐scale EM‐field sensing to obtain more information is still very challenging. Here, an adaptive information metasurface is proposed to enable intelligent sensing and wave manipulating simultaneously or more specifically, to realize intelligent target localization and beam tracking adaptively. The metasurface is composed of an array of meta‐atoms, and each is loaded with two PIN diodes and a sensing‐channel structure, for polarization‐insensitive and programmable beamforming and sensing. By controlling the state of the PIN diode, the proposed meta‐atom has 1‐bit phase response in the designed frequency band, while the sensing loss keeps higher than ‐10 dB for both “ON” and “OFF” states. Hence there is nearly no interaction between the beamforming and sensing modes. Experiments are conducted to show multiple functions of the metasurface, including intelligent target sensing and self‐adaptive beamforming, and the measured results are in good agreement with the numerical simulations and theoretical calculations.

## Graphical abstract

An adaptive information metasurface is proposed to enable intelligent sensing and wave manipulating simultaneously, or more specifically, to realize intelligent target localization and beam tracking adaptively. Experiments are conducted to show multiple functions of the metasurface, including the intelligent target sensing and self‐adaptive beamforming, and the measured results are in good agreement with the numerical simulations and theoretical calculations.

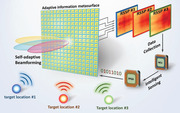

## 1 Introduction

Information metasurfaces, 2D artificial structures consisting of subwavelength elements developed from digital coding metamaterials, garnered significant attention due to their ability to flexibly control electromagnetic (EM) wave properties and link the physical world to the digital world.^[^ ^1^, ^2^, ^3^, ^4^, ^5^, ^6^ ^]^ Early‐stage information metasurfaces with fixed coding sequences have already demonstrated the powerful capacity for EM manipulations.^[^ ^7^, ^8^, ^9^, ^10^, ^11^, ^12^, ^13^ ^]^ By loading tunable electronic components in the meta‐atom, the coding sequences of the metasurface can be edited in a programmable way, leading to dynamic and real‐time manipulations of the EM waves and direct information processing. Numerous high‐performance information metasurfaces with low cost and low profile have been presented for various intriguing applications, such as reconfigurable beamforming,^[^ ^1^, ^14^ ^]^ information entropy,^[^ ^15^, ^16^ ^]^ wireless communication,^[^ ^17^, ^18^, ^19^, ^20^, ^21^, ^22^, ^23^, ^24^ ^]^ and dynamic holography and imaging.^[^ ^25^, ^26^ ^]^

In the past few years, sensing‐enabled metasurface has become one of the steadily increasing interests as it is an essential step in the pursuit of intelligent metasurface. Intelligent metasurfaces driven by a closed‐loop system, typically including a sensing module, a microcontroller unit (MCU) loaded with specific algorithms, a control board, and a metasurface array, exhibit the capacity to achieve versatile advanced functions.^[^ ^27^, ^28^, ^29^, ^30^, ^31^, ^32^, ^33^ ^]^ Previously, the metasurfaces integrated with various sensors, including gyroscope,^[^ ^29^ ^]^ depth camera,^[^ ^34^ ^]^ visible‐light detectors,^[^ ^35^ ^]^ infrared light sensors,^[^ ^36^ ^]^ ultrasonic detectors,^[^ ^37^ ^]^ and memory material^[^ ^38^ ^]^ were proposed. However, these sensors need to be equipped with extra peripheral circuits, which limits the integration level of the metasurfaces. In addition, the forms of the sensing (gravity, light, sound, force…) and manipulation (EM waves) objects are distinguished, which limits further applications.

EM sensing has become a scientific hotspot in recent years as it enables unobtrusive and noncontactless sensing of targets.^[^ ^31^, ^39^ ^]^ Previously proposed metasurface‐assisted technology for the direction of arrival (DOA) estimation and metasurface‐assisted radar systems to sense the target objects have verified the EM sensing capacity of the information metasurface.^[^ ^40^, ^41^, ^42^, ^43^, ^44^ ^]^ Integrating various functionalities into a single platform is highly valuable in practical applications.^[^ ^45^ ^]^ This is particularly true for achieving multiple functions simultaneously instead of in a time‐division switching manner.^[^ ^46^, ^47^, ^48^ ^]^ Therefore, the next stage goal is to develop a device/system that can simultaneously perform EM sensing and manipulation. To this end, some metasurfaces with EM sensors placed among the meta‐atoms array were proposed to recognize the basic properties of EM waves (e.g., frequency,^[^ ^49^ ^]^ amplitude,^[^ ^50^ ^]^ and polarization^[^ ^51^ ^]^) and manipulate the EM wavefronts simultaneously. However, all these works only deploy a few EM sensors among the elements array to recognize the EM states at a few fixed spatial points. Compared with the 2D arranged metasurface elements, the EM sensors are still in 0D or 1D scale, leading to incomplete EM sensing with limited information, thereby limiting the intelligence level of the metasurface. Furthermore, well‐performed metasurface hardware is needed for the research on the emerging integrated and sensing communication technology, since most of the works only contribute to the theoretical derivations and intend to have experimental verification.^[^ ^52^, ^53^, ^54^ ^]^ Thus, the information metasurface hardware that can sense more complex and detailed EM field information while controlling the scattering in a programmable way is in imminent demand.

In this article, we propose a new strategy for dual tasks of intelligent target sensing and self‐adaptive beamforming using the metasurface formed by meta‐atoms with simultaneous functions of programmable reflections and sensing (SFPRS), in which each meta‐atom acts as a polarization‐insensitive EM power sensor and 1‐bit EM phase modulator. The EM waves radiated from a distance can be received, sensed, and modulated to redirect to a preset direction adaptively. The individually addressable reconfigurability of the meta‐atoms enhances the EM sensing capacity and enables flexible controls of the scattering beam. Integrated with a closed‐loop system, the adaptive information metasurface can realize specified functions without the aid of human beings. As a proof of concept, the information metasurface is designed, fabricated, and measured. In the experiments, the target locations are estimated accurately, and the scattering beams are adaptively controlled based on the sensing results, where the measured and simulated deflection angles are in good agreement.

## 2 Principle and Results

**Figure 1:**
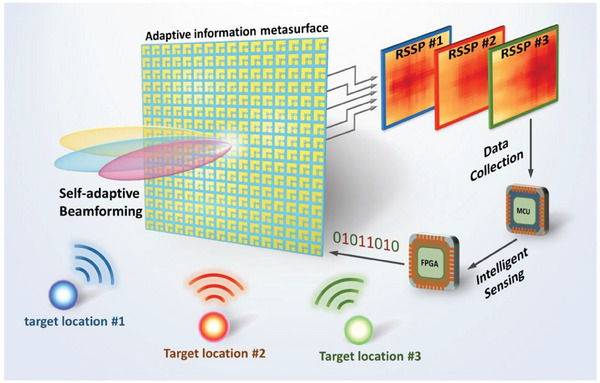
Diagram illustration of the proposed information metasurface, which includes PIN diodes and RMS power detectors that are incorporated into an elaborately designed metasurface structure. The meta‐atom serves as a 1‐bit reflection phase modulator and an RF power sensor simultaneously. The MCU can analyze the collected RSSP data provided by the sensing module for intelligent sensing, and the metasurface is encoded to realize self‐adaptive beamforming, in which the coding pattern is generated based on the estimated target location.

**Figure** **1** shows a schematic of the proposed adaptive information metasurface, which consists of an array of meta‐atoms without peripheral sensors. Each meta‐atom can be regarded as a dual‐functional device composed of a programmable reflection module and a sensing module. The principle of SFPRS is that under the illuminations of near‐field power sources (targets, marked in different colors) in the upper half‐space (z  >  0), the meta‐atom serves as a highly efficient 1‐bit reflection‐type metasurface to reflect most of the incident waves in two digital states with 180° phase difference. On the other hand, a small portion of the incident waves is transformed into guided waves and then transmitted to the root‐mean‐square (RMS) power detector for EM power sensing. The power detector can output different voltages to the MCU according to the input power level. After collecting the received signal strength of each meta‐atom in the metasurface array, the received signal strength pattern (RSSP) in the 2D scale can be obtained, which can further be used to estimate the location of the target. With the sensing information, the field programmable gate array (FPGA) is able to perform intelligent tasks like self‐adaptive beamforming and detect the target by implementing the related coding patterns.

### 2.1 SFPRS Meta‐Atom

**Figure 2:**
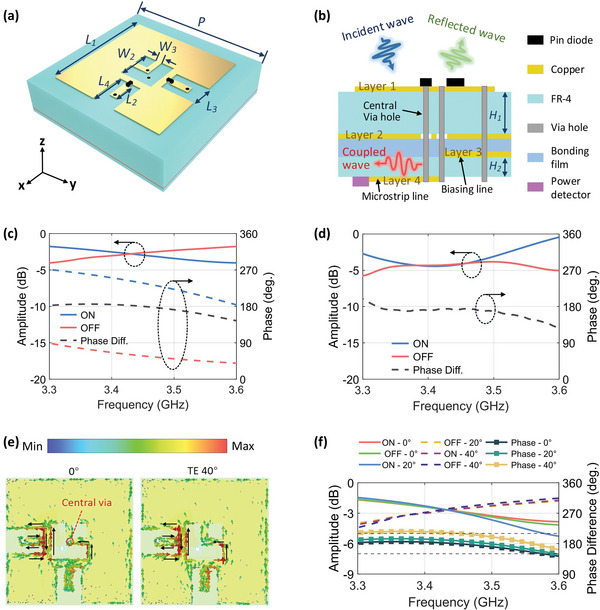
a) 3D schematic of the designed SFPRS meta‐atom. b) Side view of the SFPRS meta‐atom with an illustration of the EM‐wave routes. c) Simulated reflection amplitudes and phases of the meta‐atom. d) Measured reflection amplitudes and phases of the meta‐atom. e) Surface current distribution under the illumination of traverse‐electric (TE) polarized wave at 0° and 40°. f) Simulated reflection amplitudes and phases of the meta‐atom under TE‐polarized oblique incidence.

As sketched in **Figure** **2**a, the meta‐atom structure is shown in a 3D view. The sandwich‐like element structure is piled up using printed circuit board (PCB) technology with two substrate layers (FR‐4, the dielectric constant is 4.3, the loss tangent is 0.025), whose thicknesses are *H*_1_ = 4 mm and *H*_2_ = 4 mm, respectively. Each dielectric slab is dual‐sided copper clad and is mutually laminated by a bonding film. Namely, the meta‐atom has four metallic layers. As shown in Figure 2b, layer #1 is a reflective patch structure embedded with two PIN diodes for programmable reflections. Two PIN diodes are loaded in the gap between the central patch and two metal bars along the *x*‐ and *y*‐axes (with the anodes on the patch) to tailor two orthogonal polarized reflection phases simultaneously. Layer #2 is a metallic screen with a hole in the center that reflects most incident waves while allowing minor incident waves to transmit through the central via‐hole to the sensing module. To facilitate the PCB routing, the biasing lines and the sensing module are placed in different layers, named as layer #3 and layer #4, respectively. In layer #4, the sensing power from the via‐hole is transmitted through the microstrip line to the RMS power detector. For analysis simplicity, the RMS power detector can be approximately regarded as a 50 Ω terminal load in the full‐wave simulations. It should be noted that the central via‐hole (marked in Figure 2b) serves as a transmitting route for AC and DC simultaneously. The structural parameters are optimally set as follows: *P* = 20 mm, *L_1_* = 15.3 mm, *L_2_* = 2.4 mm, *L_3_* = 3.1 mm, *W*_1_ = 1 mm, *W*_2_ = 4.5 mm, and *W*_3_ = 0.8 mm.

The element simulation is conducted using commercial simulation software CST Microwave Studio 2019, in which unit‐cell boundary setting (see Figure 2e) is used to mimic the metasurface with infinite numbers of meta‐atoms. Figure 2c,d plot the simulated and measured amplitude and phase responses, respectively. It is observed that the simulated reflected magnitude has a peak value of −2.8 dB at 3.475 GHz. Two factors lead to non‐ideally high reflection amplitude: 1) the energy dissipation of the Fr‐4 substrate due to its high loss tangent of 0.025; and 2) the small portion of the energy absorption in the sensing channel. As a tradeoff between the fabrication cost, reflection amplitude, and sensing performance, the simulated reflection amplitude of the meta‐atom is optimized to be around −3 dB in the operating band, which is still acceptable in most cases. In the operating bandwidth of 50 MHz, the phase difference fluctuates within 180 ± 9° enabling a stable 1‐bit phase modulation. Given that the structure of the presented element is symmetrical along the *x*‐ and *y*‐axes, the reflected amplitude and phase are polarization‐insensitive. We noted that, compared with the simulation results, the measured reflected amplitude slightly drops. The error is mainly attributed to the parasitic effect of the PIN diodes that are not considered in the simulations and the error in the fabrication process.

The distributions of surface current density at incident angles of 0° and 40° are shown in Figure 2e to illustrate the working mechanism and the angular performance of the meta‐atom. It is observed that the surface currents are concentrated around the PIN diode, allowing the PIN diode to control the surface currents effectively to meet the 1‐bit reflection phase requirement. Due to the similarity of the current distributions at 0° and 40°, the EM response of the meta‐atom is stable within the angular range from 0° to 40°. Specifically, the phase intervals between the two digital states stay within ± 30° at the incident angles of 0°, 20°, and 40°, as demonstrated in Figure 2f. In addition, the amplitudes are stably higher than −3 dB near 3.45 GHz. Therefore, the proposed meta‐atom is qualified for wide‐angle applications.

**Figure 3:**
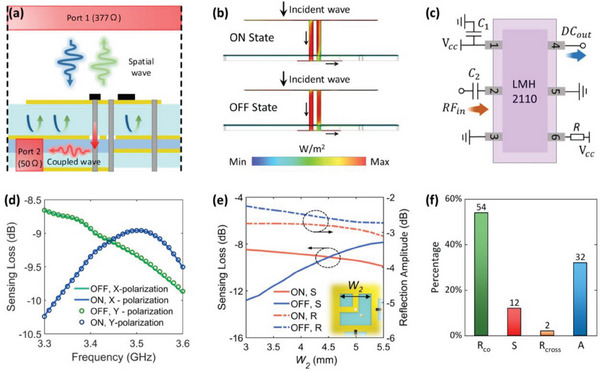
a) Schematic diagram of the configuration in the full‐wave simulation, including boundary condition and wave port. b) Side view of the power flow distributions with two digital states under normal incidence. c) Sketch of the configuration of the LMH2110 RMS power detector. d) Simulated sensing losses of the meta‐atom with two digital states in the *x*‐ and *y*‐polarizations. e) Parametric analysis of *W_2_* to reveal the mechanism of keeping the sensing loss under two digital states the same. f) Incident energy transforms to copolarization reflection *R*_co_, sensing loss *S*, cross‐polarization reflection *R*_cross_, and absorption *A*, where 1(100%) = *R*_co_ + *S* + *R*_cross_ + *A*. The percentage of each energy part is demonstrated in Figure 3f.

To inspect the EM sensing performance of the meta‐atom, we define the S‐parameter from port #1 (Floquet port that illuminates plane wave) to port #2 (50 Ω discrete port at the end of the sensing module) as sensing loss to evaluate the EM sensing quantitatively. As shown in **Figure** **3**a, a small portion of the EM wave radiated from port #1 can be received by the meta‐atom structure and transmitted to the RMS power detector with 50 Ω input impedance. Figure 3b plots the section view of the power flow density along the surface of the meta‐atom. The power transmitting path from top to bottom is clearly seen. The coupled wave at the bottom layer will finally collected by the RMS power detector chip (LMH 2110), whose configuration is shown in Figure 3c. Within the dynamic range, the DC output voltage (*DC_out_*) relates linearly to the RF input power (*RF_in_*) in dBm. The simple corresponding relationship between *DC_out_* and *RF_in_* facilitates the MCU to retrieve the RSSP. As depicted in Figure 3d, the sensing loss is higher than −10 dB for both “ON” and “OFF” states, which enables the meta‐atom to flexibly sense the EM powers in different digital states. By tuning the parameter *W_2_*, the sensing loss at two digital states can be almost the same at 3.45 GHz, as illustrated in Figure 3e. In Figure 3f, it is clearly shown that the illuminated energy is transformed into different energy forms, including co‐polarization reflection (*R_co_*), sensing loss (*S*), cross‐polarization reflection (*R_cross_*), and absorption (*A*). The percentage of each energy form is also given, with the total sum being 1 or 100%, as *R_co_* + *S* + *R_cross_* + *A* = 1.

### 2.2 Task I: Intelligent Target Sensing

As discussed in the last section, the designed SFPRS meta‐atom can sense the amplitude of the illuminated EM waves. More importantly, the proposed metasurface is capable of sensing complex EM information by collecting the RSSP on a 2D plane. This is because the large‐scale fields allow for sensing the amplitude of each meta‐atom and the amplitude difference between adjacent meta‐atoms, enabling more information acquisition. For example, though the sensing loss is insensitive to the polarization of EM waves, the orthogonal polarizations can still be recognized under periodic coding sequences of “010101…” (see Section S1, Supporting Information). To fully demonstrate the strong EM sensing capacity, a method for intelligent target sensing aided by the proposed information metasurface is presented below.

The traditional indoor target localization method based on processing signal strength information at multiple base stations is one of the most popular and simplest methods.^[^ ^55^, ^56^ ^]^ Inspired by this, the proposed metasurface composed of numerous meta‐atoms, each functioning as a base station, is expected to realize target localization and intelligent beamforming in a highly integrated and compact architecture. It can be inferred that the RSSP under the illumination of the target's RF signal is a function of the target's location, as shown in Figure 1. Although collecting a sufficiently large number of data to record the relationship between the target location and RSSP is theoretically viable, the data collection process is time‐consuming and needs to be repeated when the EM environment changes. Therefore, we intend to present a new target localization strategy by only collecting limited RSSPs. In the experiment, RSSPs at fifteen anchor points are measured in total for the target sensing.

#### 2.2.1 Theoretical EM Model

**Figure 4:**
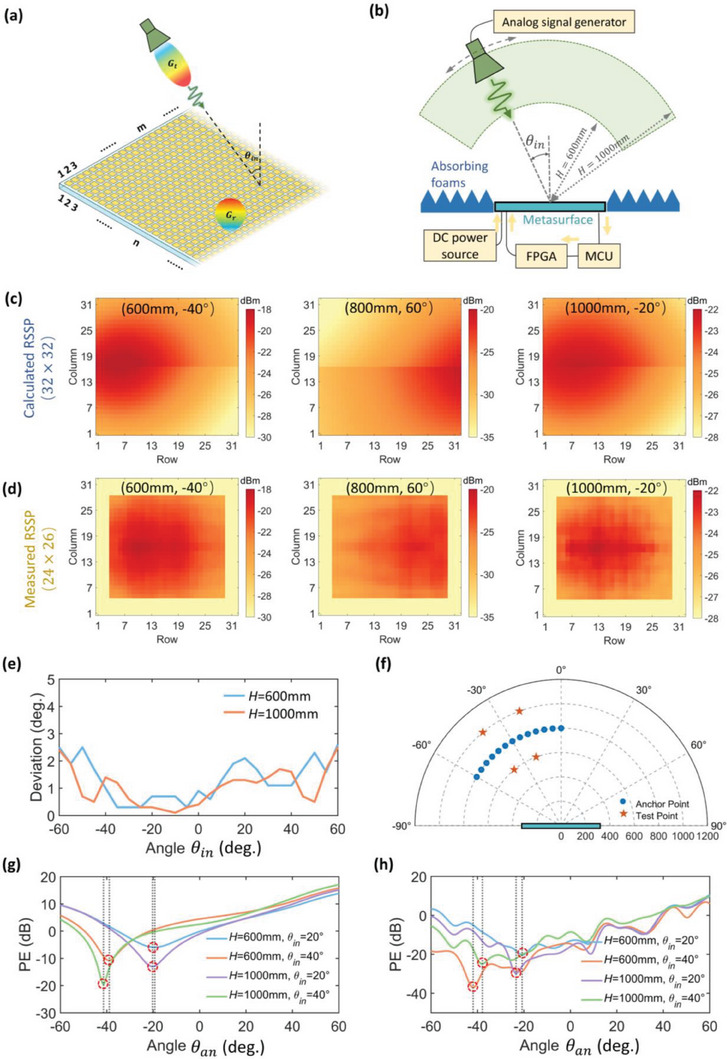
Theoretical analyses of the proposed near‐field DNS estimation and the experimental verification at 3.45 GHz. a) Theoretical EM model of the target and the information metasurface. b) Illustration of the experimental setup for DNS estimation. c) Calculated RSSPs under the target locations of (600 mm, −40°), (800 mm, 60°), and (1000 mm, −20°). d) Measured RSSPs under the target locations of (600 mm, −40°), (800 mm, 60°), and (1000 mm, −20°). e) The angle deviations between the actual incident angle and the estimated angle at different test angles *θ_i_*. f) Locations of the anchor points and test points during DNS estimations. g) Simulated pattern errors at four test points. h) Measured pattern errors at four test points.

We assume no significant obstacle is between the metasurface and the target. Thereby, a theoretical EM model with free‐space path loss is proposed to calculate the RSSP quickly. As depicted in **Figure** **4**a, a planar metasurface composed of 32 × 32 elements with a period size *D* = 20 mm is illuminated by the feeding horn at the incident angle *θ_in_* with the distance *H* away from the metasurface center. The Mth row and *N* column element of the RSSP matrix (i.e., *RSSP* (*m*, *n*)) can be theoretically calculated using the Friis transmission equation:

(1) $$\begin{array}{ccc} RSSP\;\left(m,n\right) & = & \frac{P_{r}\left(m,n\right)}{P_{t}}=\;20\log_{10}\frac{\lambda }{4\pi R\left(m,n\right)}+G_{r}\left(\theta_{r},\phi_{r}\right)\\ & & +\;G_{t}\left(\theta_{t},\phi_{t}\right)\;\left(\mathrm{dB}\right) \end{array}$$

where *P*_t_ is the transmitting power of the target. *P*_r_ (m, n) is the received power of the Mth row and Nth column of the metasurface. *R*(*m*, *n*) is the distance between the feeding horn and each element. Since the EM sensing and radiation are reciprocal, the far‐field pattern of the single element excited by port #2 is obtained by full‐wave simulation, i.e., *G_r_* (*θ_r,_ φ_r_*). Similarly, the far‐field pattern of the target *G_t_* (*θ_t,_ φ_t_*) is simulated. We note that although the distance between the feeding horn and the metasurface does not fully meet the far‐field condition specified by the Friis transmission equation, our theoretical model can still yield precise outcomes quickly (see Section S2, Supporting Information). The proposed EM model solves the difficulty in full‐wave simulation of an electrically large metasurface with an electrically thick profile due to space‐fed configuration, thereby greatly reducing the computational burden and consumed time. The calculated RSSP can be used as “course data” to inspect and verify the performance of the proposed estimation strategy preliminarily.

#### 2.2.2 Estimation for the Direction of a Near‐Field Source

The experimental setup to measure the RSSP of the designed metasurface is shown in Figure 4b, where the broadband double‐ridged horn antenna is used as the transmitting antenna (see Section S3, Supporting Information). The calculated and measured RSSP with the transmitting antenna at the locations of (600 mm, −40°), (800 mm, 60°), and (1000 mm, −20°) under 20 dBm input power level are shown in Figure 4c,d, respectively. The slight discrepancy between the calculated RSSP and the measured RSSP can be attributed to the fact that the theoretical EM model does not take into account the mutual coupling between the metasurface elements, and the actual received signal strength is also affected by fabrication errors. From both the calculated and measured results, there appears to be a clear correlation between *θ*_in_ and RSSP. This mapping relationship provides a useful strategy for estimating the direction of a near‐field source (DNS). The strategy can be described as follows.

First, we obtain the RSSPs at several anchor points as prior information. As shown in Figure 4f, RSSPs of 13 anchor points (*RSSP θ*_an_ anchor*, θ*_an_ = −60°, −55°,…, 0°) ranging from −60° to 0° with a step size of 5° are calculated, where the distance *H* is set to be 800 mm as a typical near‐field distance. The *RSSP θ_an_ anchor* from 0° to 60° can be obtained by flipping the corresponding *RSSP θ*_an_ anchor from −60° to 0° directly since the far‐field pattern of the metasurface element is nearly symmetric. Second, acquire the RSSP of the target (*RSSP_test_*) under test. Next, compare the normalized RSSP of the target *RSSP θ*_an_ test(norm) with all *RSSP θ*_an_ anchor (*θ*_an_ = −60°, −55°,…, 60°) and calculate the pattern error (*PE*) correspondingly. To normalize *RSSP_test_*, a constant power level is added to *RSSP*_test_ to ensure its peak value is equal to the peak value of *RSSP θ*_an_ anchor, which can offset the power level deviation caused by the fluctuation of *H*. Pattern error, is defined as the difference level between the *RSSP θ*_an_ anchor and the *RSSP_target_*. This difference is calculated using the following formula:

(2) $$PE\left(\theta_{an}\right)\;=\;\frac{\sum_{M\;=Row_{s}\;}^{Row_{e}}\sum_{N\;=Column_{s}\;}^{Column_{e}}K\left(M,N\right)\;{\left(RSSP_{target\left(norm\right)}^{\theta_{an}}\left(M,N\right)-RSSP_{anchor}^{\theta_{an}}\left(M,N\right)\right)}^{2}}{\sum_{M\;=Row_{s}\;}^{Row_{e}}\sum_{N\;=Column_{s}\;}^{Column_{e}}K\left(M,N\right)}$$

The specific description of pattern error *PE* and the definitions of each parameter in Equation (2) are illustrated in Section S4 (Supporting Information), in detail.

As discussed above, *PE* corresponding to each anchor point can be calculated after comparing the *RSSP*_target_ of the target with all *RSSP θ*_an_ anchor using Equation (2). Similar to the spectrum peak search in the classic DOA estimation, e.g. MUSIC algorithm, in our strategy, the angle *θ*_an_ that has the lowest *PE* is the estimated DNS (i.e., *θ*_estimated_). This is because the lowest *PE* denotes the highest similarity between *RSSP*_target_ and *RSSP θ*_an_ anchor at *θ*_estimated_. To improve the DNS estimation precision limited by the 5° step size of the anchor, the *PE* against incident angle is fitted using cubic spline interpolation to allow *θ*_estimated_ to be continuous instead of discrete. To further improve the DNS estimation accuracy in a near‐field area, parameter optimization can be utilized. We take the near‐field area within an angular range from *θ*_in_ = −60° to 60° and a distance range from *H* = 600 mm to 1000 mm as an example. The optimization goal is to minimize the absolute deviations (*θ*_deviation_) between the actual angles and the estimated angles under different illuminated angles at *H* = 600 mm and 1000 mm. The final optimized parameters are shown in the Supporting Information.

The absolute deviations *θ*_deviation_ within the angular range from −60° to 60° are shown in Figure 4e, in which the maximum absolute deviation is 2.4°, indicating good DNS estimation accuracy. Although the results presented in Figure 4e rely on the calculated RSSP, the theoretical feasibility of the proposed strategy for DNS estimation has been well demonstrated. To provide a more comprehensive understanding of the DNS estimation performance, Figure 4g,h displays the PE curves for target locations at (600 mm, −40°), (600 mm, −20°), (1000 mm, −40°), and (1000 mm, −20°), respectively. In Figure 4f, the thirteen anchor points tested previously, as well as the four testing points mentioned above, are also depicted. It is found that the *θ*_estimated_ obtained from the calculated and measured data are very close to the actual incident angles. This encouraging result confirms the feasibility and accuracy of the proposed DNS estimation numerically and experimentally. We mention that the accuracy of the proposed DNS estimation method is not limited to a few points within the near‐field area. In fact, the method can provide accurate DNS estimation for multiple points within the near‐field region, see Section S5 (Supporting Information.)

As a preliminary proof‐of‐concept, the measured *PE* curves in Figure 4h exhibit undesired fluctuations due to inconsistencies in electronic devices, fabrication errors, and measurement errors, which may increase the rate of erroneous DNS estimation when subjected to external interference. Analytical results show that the strategy has good anti‐interference ability when interfered with an external power source. However, when the interference is very considerable, such as the scattering wave from a human body standing very close to the metasurface, the measured RSSP is deformed and distorted greatly due to the multipath effect, leading to less accurate results (see Section S6, Supporting Information). However, it should be noted that when the target is in the near‐field of the metasurface, it is possible to implement an EM environment with minimized interference, allowing our fabricated metasurface to remain applicable in most cases.

#### 2.2.3 Near‐Field EM Ranging

According to the proposed theoretical EM model, the received signal strength of the meta‐atoms is expected to relate linearly to 1 H*^−^* ^2^ as described in Equation (1). In the EM ranging process, it is important to consider that the signal strength may vary among different targets. As such, it is necessary to obtain prior knowledge of the targets' signal strength to normalize the RSSP. It should be noted that the received signal strength is not only influenced by the distance between the target and metasurface, but also by the orientation of the target's transmitting antenna and the incident angle of the RF signal. This is due to the angular dispersion of far‐field patterns of the target and the metasurface element, which can affect the accuracy of the received signal strength. To simplify the EM ranging experiment, we always direct the main lobe of the transmitting antenna toward the center of the metasurface. However, if the target's transmitting antenna is omnidirectional, such as a dipole antenna, the impact of the target's orientation will be diminished, and the transmitting antenna may not need to be directed toward the metasurface center. Furthermore, we will present a method to compensate for the ranging error caused by the oblique incidence of the target's signal.

**Figure 5:**
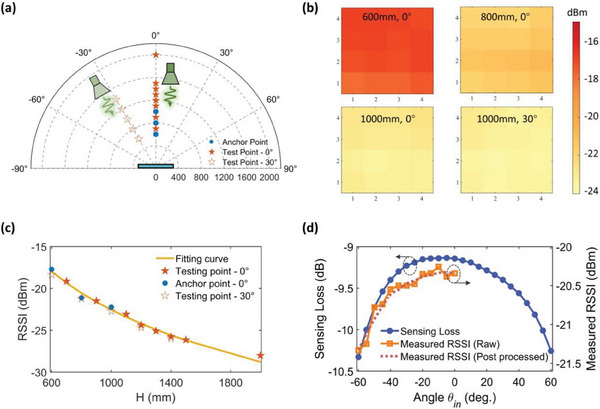
Near‐field EM ranging performance at 3.45 GHz. a) Locations of transmitting antennas with the anchor points marked in blue dots and the test points marked in red pentagrams during the EM ranging. b) Received signal strength of the central 4  ×  4 elements of the metasurface under the target locations of (600 mm, 0°), (800 mm, 0°), (1000 mm, 0°), and (1000 mm, 30°). c) A curve illustrating the relationship between the RSSI and the distance *H* is generated by fitting the measured RSSIs at the anchor points. RSSIs at the testing points are very close to the fitting curve, indicating good EM ranging performance. d) Simulated sensing losses and measured RSSIs against the incident angle at distance *H* = 800 mm. The orange solid line is the raw measured RSSIs, and the reddish‐brown dotted line denotes the post‐processed results.

To perform real‐time EM ranging, we first collect the measured RSSP at three anchor points along the normal direction of the metasurface with distances of 600 mm, 800 mm, and 1000m, as shown in **Figure** **5**a. Since the RSSP at a distance of 800 mm has already been measured as an anchor point during the previous DNS estimation process, only two additional anchor points need to be measured. We use the average received signal strength of the central 4 × 4 elements of the metasurface as the received signal strength indicator (RSSI) for EM ranging. Figure 5b intuitively shows the measured received signal strength of the central 4 × 4 elements when the target is at different locations. Based on the measured results at the three anchor points, we fit the RSSI in decibels with respect to the distance H using the form of *RSSI* = *A* log_10_ *H* + *B*, where *A* and *B* are fit parameters optimized to −20.7 and 39.5, respectively.

Next, we measured the RSSIs at eight test points with *H* of 700, 900, 1100, 1200, 1300, 1400, 1500, and 2000 mm for EM ranging. As shown in Figure 5c, it can be observed that the RSSIs at the test points within *H* from 600 to 1500 mm are very close to the fitting curves, indicating slight estimation errors. The deviation at *H* = 2000 mm can be attributed to the increased signal‐to‐noise ratio since the received signal strength becomes weak at a far distance. Figure 5c also compares the measured RSSIs at the normal incidence and the incidence of 30°. It is found that the RSSIs fluctuation is slight against the incident angles but still affects the estimation accuracy. Fortunately, the fluctuation is a system error caused by the angular dispersion of the far‐field pattern of the meta‐atom, which can be offset by proper calibration. To interpret this point, the simulated sensing loss of the meta‐atom and the measured RSSIs of the *RSSP*_anchor_ at 3.45 GHz are shown in Figure 5d. The raw measured data and the data postprocessed by smoothing and interpolation are denoted by the orange solid line and the reddish‐brown dotted line, respectively. It is observed that the simulated sensing loss and the measured RSSIs have a similar tendency against incident angles. Therefore, the RSSIs of the previously measured thirteen *RSSP*_anchor_ for DNS estimation can be used as calibration to refine EM ranging under oblique incidence. To be specific, the fit parameter *B* is dependent on the estimated angle *θ*_estimated_. When *θ*_estimated_ is in the range of −2.5° to 2.5° (normal incidence), *B* is set as 39.5, as discussed above. If θ_estimated_ is in another angular interval, *B* should be adjusted according to the relationship plotted in Figure 5d.

#### 2.2.4 Near‐Field Target Localization

**Figure 6:**
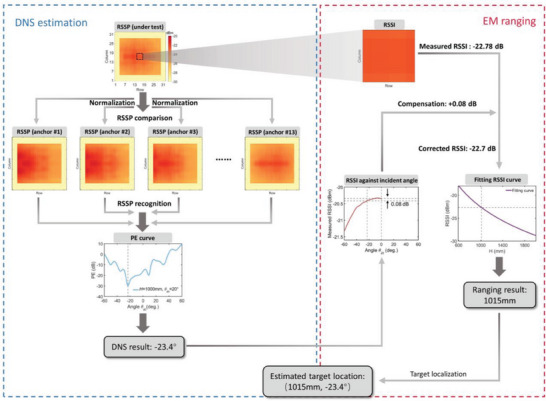
Flowchart that describes the proposed target localization strategy step by step. Estimating the position of the target object at the location of (1000 mm, −20°) is used as an example to clarify the processing procedure.

In the last section, good performance of DNS estimation and EM ranging have been demonstrated, laying the foundation for intelligent target sensing, or more specifically, target localization. For low power consumption, each meta‐atom is set to an “OFF” state during the target sensing process. The MCU will obtain the final estimated target location through the built‐in calculation method discussed above. The flowchart depicted in **Figure** **6** outlines the step‐by‐step procedure of the target localization strategy in detail by using an example.

**Figure 7:**
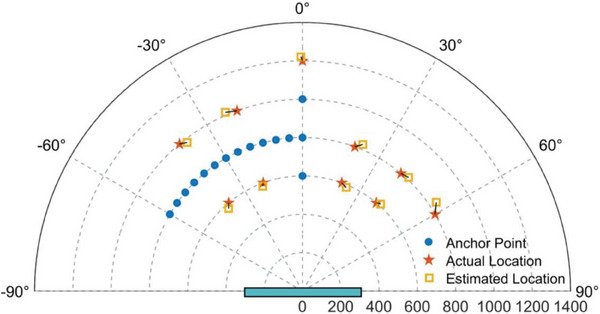
Experimental verification of the near‐field target localization. The locations of the anchor points and the actual locations of the transmitting antennas are depicted in blue dots and red pentagrams, respectively. The estimated target locations, marked in the orange squares, are linked to the actual locations of the transmitting antenna by a black line.

To thoroughly demonstrate the target localization performance of the fabricated adaptive information metasurface, RSSP at ten different target positions is measured for testing. **Figure** **7** depicts the anchor points, the actual target locations, and the estimated locations in blue dot, red pentagram, and orange square, respectively. The location estimation error is denoted by the black dashed lines. Detailed measured data are listed in **Table** **1**, in which the worst DNS estimation error and EM ranging error are 3.8° and 38 mm, respectively. As a proof‐of‐concept, the measurement results are encouraging enough to verify the proposed intelligent target sensing strategy. Although the optimal sensing coverage is still limited in our presented experiment, we remark that the sensing coverage can be expanded if more RSSP data at different anchor points are measured and collected.

**Table 1:** Comparison between the actual target location and the estimated location.

Sequence	Actual location	Estimated location	Sequence	Actual location	Estimated location
#1	600 mm, −40°	578 mm, −41.9°	#6	800 mm, 40°	811 mm, 43.1°
#2	600 mm, −20°	584 mm, −20.8°	#7	800 mm, 60°	838 mm, 56.5°
#3	600 mm, 20°	586 mm, 22.9°	#8	1000 mm, −40°	981 mm, −37.9°
#4	600 mm, 40°	610 mm, 42.1°	#9	1000 mm, −20°	1015 mm, −23.4°
#5	800 mm, 20°	826 mm, 22.6°	#10	1200 mm, 0°	1221 mm, −0.4°

### 2.3 Task II: Self‐Adaptive Beamforming

**Figure 8:**
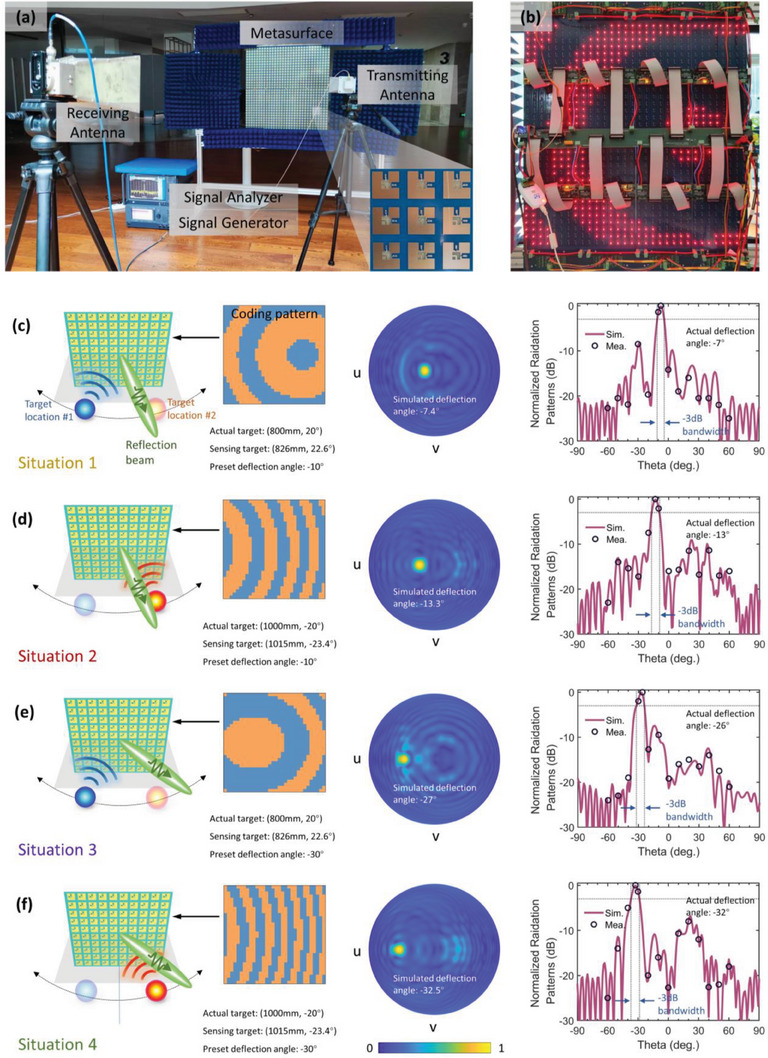
Experimental verifications of intelligent beamforming in four situations. a) Photograph of the experimental setup with the zoomed‐in view of the fabricated metasurface at the bottom‐right corner. b) Back view of the fabricated metasurface. c–f) In four specific situations: c) situation #1, d) situation #2, e) situation #3, f) situation #4, the generated coding patterns, the simulated 2D far‐field gains in linear scale, and comparison between the measured and the simulated radiation patterns are shown in the left, middle, and right panels, respectively.

By continuously estimating the target location and receiving feedback in real‐time, the adaptive information metasurface is able to intelligently manipulate the EM waves. Based on the estimated target location, the metasurface is expected to steer the reflection beam to different preset directions automatically and self‐adaptively. To further validate this point, the experiment is conducted in a real indoor environment, as shown in **Figure** **8**a. The 2D schematic diagram of the experimental scenario is shown in Section S8 (Supporting Information.) The fabricated prototype consisting of 32 × 32 elements is fixed at a holder using bolts and nuts. The broadband double‐ridged horn antenna aligned with the analog signal generator (Keysight E8RSSP7D) radiates spherical waves to mimic the target under test. The reflection wave is received by the horn antenna connected with the signal analyzer (Keysight N9040B) to measure the far‐field pattern of the metasurface. As illustrated in Figure 8b, the closed‐loop system including the controlling circuit network, MCU, and FPGA is placed at the back of the metasurface, where the surface‐mounted LEDs serially connected in the biasing line are used to display the coding pattern in real‐time.

During the experiment, we moved the transmitting antenna (near‐field target) around the metasurface. The MCU has inputted some preset reflection directions, allowing the coding patterns to be automatically generated and switched in real time. Measured results show that the deflection angles of the reflection beams are consistent with the preset ones. To better illustrate this, the far‐field results in four typical situations are simulated and measured. In situations #1 and #2, the target locations are respectively at (800 mm, 20°) and (1000 mm, −20°), while the preset deflection angle in both situations is 10°. Similarly, in situations #3 and #4, the target locations are respectively at (800 mm, 20°) and (1000 mm, −20°), while the preset deflection angle in both situations is set to 30°. The sensing target location in situations #1 and #3 is (826 mm, 22.6°), and the sensing target location in situations #2 and #4 is (1015 mm, −23.4°), as listed in Table I.

The generated coding patterns in the four situations (situations #1 to #4) are shown in the left panel in Figure 8c–f. Blue and orange pixels of the coding pattern represent the two designed phase responses listed in Figure 2c,d. The simulated 2D far‐field result in the upper space is given in the middle panel. The comparison between the simulated and measured radiation pattern is in the right panel. In situations #1 to #4, the simulated main beams are directed to 7.4°, 13.3°, 27°, and 32.5°, respectively. Due to the experimental limitations, it is hard to measure the far‐field pattern of the metasurface by placing it on a rotatable platform with a feeding horn antenna. However, since there is little difference between the main beam directions in the near‐field and far‐field, the radiation pattern of the metasurface can still be measured discretely by placing a receiving horn antenna 2 m away from the metasurface center at −60° to 60° with an increment of 10°. The measured radiation patterns are normalized to the strongest received amplitudes, which occur at angles *θ* = 7°, 13°, 26°, and 32°, respectively. Good agreement between the simulation and measurement results can be observed. The slight deviation between the actual deflection angle and the desired angle is attributed to the estimation error of the target location and the slight phase error of the meta‐atom. Considering that the preset directions are still within the −3 dB bandwidth of the reflection beam in all four situations, the accuracy of the self‐adaptive reflection beam control is still acceptable in many cases, indicating the good performance of the designed metasurface and the effectiveness of the proposed strategy for the intelligent sensing and beamforming.

## 3 Discussion

The working mechanism of the proposed SFPRS meta‐atom shares some similarities with the radiation‐reflection‐switching metasurface,^[^ ^57^ ^]^ as both involve radiation/sensing and reflection functions. The difference is that the radiation‐reflection‐switching metasurface needs switching components to alternate between the two functions, while the proposed SFPRS meta‐atom can perform the two functions simultaneously at the cost of low sensing loss (≈−10 dB). Each meta‐atom serves as a base station that can receive, sense, modulate, and reflect the EM waves radiated from a distance. RSSPs at fifteen anchor points are measured as prior data to match the real environment to obtain optimal sensing performance. Pattern recognition method is utilized to estimate accurate DNS, which has anti‐interference performance to a certain degree. The RSSI‐based ranging method is naturally susceptible to interference, but we remark that the stability and accuracy can be improved further by adopting the advanced filtering algorithm.^[^ ^58^, ^59^ ^]^ The closed‐loop system is integrated into the metasurface to control the sensing and reflection module intelligently. In the experiment, the ability of adaptive beamforming is chosen as a typical function to demonstrate the intelligence of the metasurface.

The phenomena of intelligent sensing and beamforming are well verified, but we mention that the beamforming ability can be further improved by using an SFPRS meta‐atom with more phase bits to achieve more intriguing phenomena, such as beam steering with low side‐lobe, generating OAM beam and holography. Adopting a multilayer structure to decouple the functionalities of EM reception/reflection, phase shift, EM sensing, DC output lines, and biasing lines is a good solution, but the uniformity of the sensing losses under different phase states, low fabrication cost, and easy integration seem to be predictable challenges.

## 4 Conclusion

We proposed an adaptive information metasurface to achieve two tasks simultaneously, target localization and intelligent beamforming. A metasurface array with individually addressable and polarization‐insensitive meta‐atom, which acts as a 1‐bit reflection phase modulator and the power detector, is simulated, designed, and fabricated. An experiment on near‐field DNS estimation is first conducted, which utilizes the RSSP recognition strategy to estimate the DNS. Furthermore, the performance of the EM ranging and the final target localization are demonstrated successively, showing accurate target sensing within a specified near‐field area. Benefiting from the target sensing capacity, intelligent beamforming can be established stably and accurately. Overall, the concept of dual‐task intelligent sensing and beamforming is verified at the physical layer level. It is a step forward in the pursuit of metasurface intelligence and opens up an avenue for diverse application fields, such as integrated sensing and communication, remote sensing, and intelligent sensors.

## Conflict of Interest

The authors declare no conflict of interest.

## Supporting information

Supporting Information

## Data Availability

The data that support the findings of this study are available from the corresponding author upon reasonable request.
