# Supplementary material for: Simultaneously Intelligent Sensing and Beamforming Based on an Adaptive Information Metasurface
Source: Adv Sci (Weinh). 2023 Dec 8;11(7):2306181. doi: 10.1002/advs.202306181 (PMC10870054; doi:10.1002/advs.202306181)
Supplement: Supplementary file 1 — Supporting Information [file ADVS-11-2306181-s001.pdf]

ADVANCED SCIENCE
Open Access

Supporting Information

for *Adv. Sci.*, DOI 10.1002/advs.202306181

Simultaneously Intelligent Sensing and Beamforming Based on an Adaptive Information Metasurface

Rui Zhe Jiang, Qian Ma, Ze Gu, Jing Cheng Liang, Qiang Xiao, Qiang Cheng* and Tie Jun Cui**

Supporting Information for

Simultaneously Intelligent Sensing and Beamforming Based on an Adaptive Information Metasurface

Rui Zhe Jiang, Qian Ma, Ze Gu, Jing Cheng Liang, Qiang Xiao, Qiang Cheng* and Tie Jun Cui**

Rui Zhe Jiang, Qian Ma, Ze Gu, Jing Cheng Liang, Qiang Xiao, Prof. Qiang Cheng and Prof. Tie Jun Cui

State Key Laboratory of Millimeter Waves, Southeast University, Nanjing 210096, China

Institute of Electromagnetic Space, Southeast University, Nanjing 210096, China

E-mail: maqian@seu.edu.cn, qiangcheng@seu.edu.cn and tjcui@seu.edu.cn

The Supporting Information includes:

S1. Method for Polarization Recognition

S2. Comparison between the Calculated and Simulated Results

S3. Transmitting Antenna Used in the Experiment

S4. Description of the Defined Pattern Error

S5. *PE* at Other Points Within the Specified Area

S6. Assessment of the anti-interference ability

S7. 2-D schematic diagram of the experimental scenario

S1. Method for Polarization Recognition

The sensing loss of the proposed SFPRS meta-atom is designed to be insensitive to polarization to avoid polarization mismatch. However, in some cases, recognition of the polarization of the illuminated wave is still important. Hence, we propose a method for polarization recognition by encoding the metasurface with the coding sequence of "010101…", to break the original boundary conditions of all "0" or all "1" and allow the metasurface array to be sensitive to polarization. Figure S1a shows the configuration of the metasurface array, where "unit 1" refers to the "ON" state of the PIN diodes and "unit 0" refers to the "OFF" state of the PIN diodes. Two adjacent meta-atoms of "0" and "1" digital states are simulated under the unit cell boundary to mimic the metasurface array under the "010101…" coding sequence, as illustrated in Figure S1b. Figure S1c shows the simulated sensing loss of the meta-atoms in "0" and "1" digital states under x- and y- polarizations. Distinguished sensing loss under two orthogonal polarizations can be observed, which can be used as an indicator to recognize the polarization of the illuminated wave.

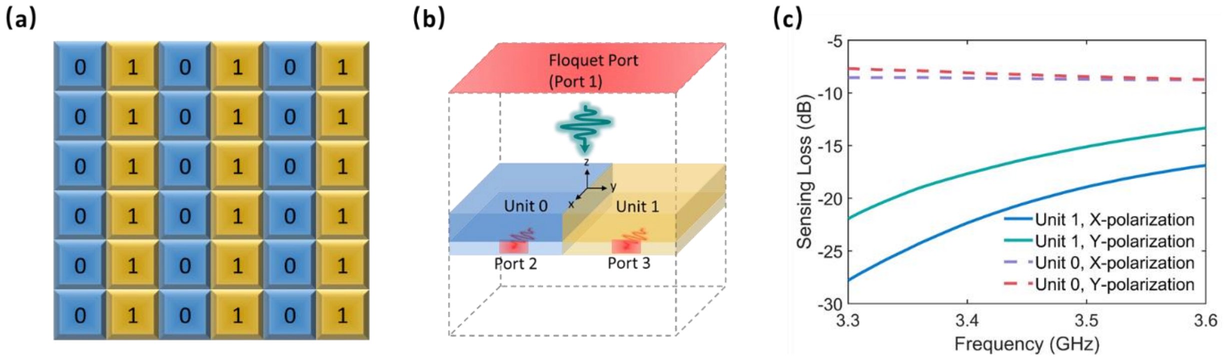

Figure S1. (a) Configuration of the metasurface array under the coding sequence of "010101…". (b) Illustration of the unit cell boundary condition in the simulation. (c) Simulated sensing loss of the meta-atoms in "0" and "1" digital states under x- and y-polarizations.

S2. Comparison between the Calculated and Simulated Results

Our proposed EM model uses the Friis equation to calculate the RSSP, which requires the far-field condition to be satisfied between the transmitter and receiver. However, in our model, the distance *H* between the transmitting antenna and the metasurface center does not strictly meet the far-field condition. To investigate the effect of this condition mismatch, we compared the simulated and calculated surface power density under the illumination of a transmitting antenna with 1 watt output power level, as shown in Figure 2S. The small differences between the simulation and calculation results indicate that our proposed calculation method is still applicable despite the condition mismatch.

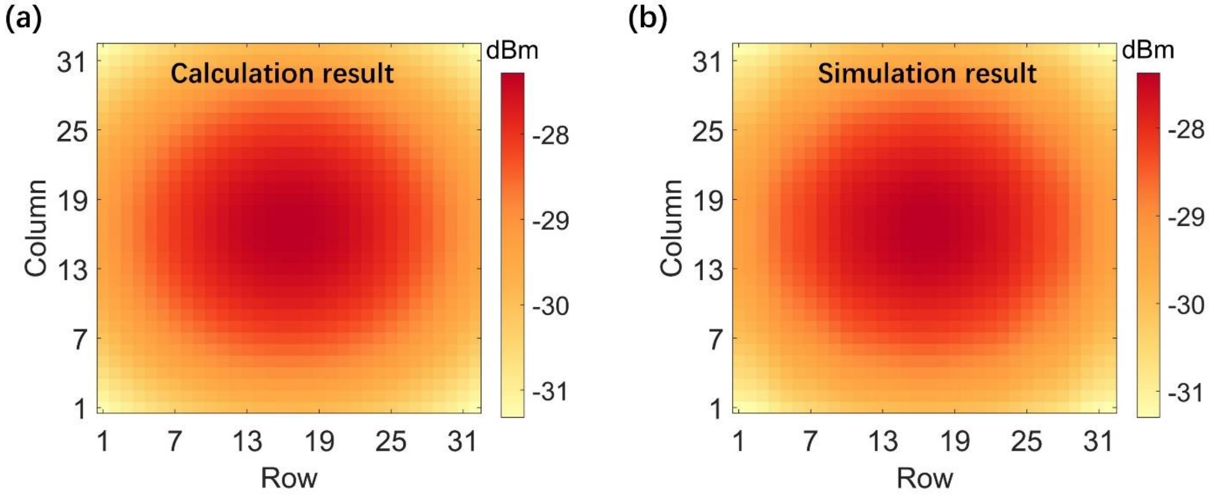

Figure S2. The comparison between the calculated surface power density and the simulated one under the normal incidence of the transmitting antenna with the distance H = 800 mm away from the RIS center. (a) calculation result. (b) simulation result.

S3. Transmitting Antenna Used in the Experiment

In our experiment, the broadband double-ridged horn antenna is used as the power source to mimic the target's transmitting antenna. The structure and the geometry parameters of the horn antenna are shown in Figure 3Sa,b. Figure 3Sc illustrates the far-field pattern of the horn antenna.

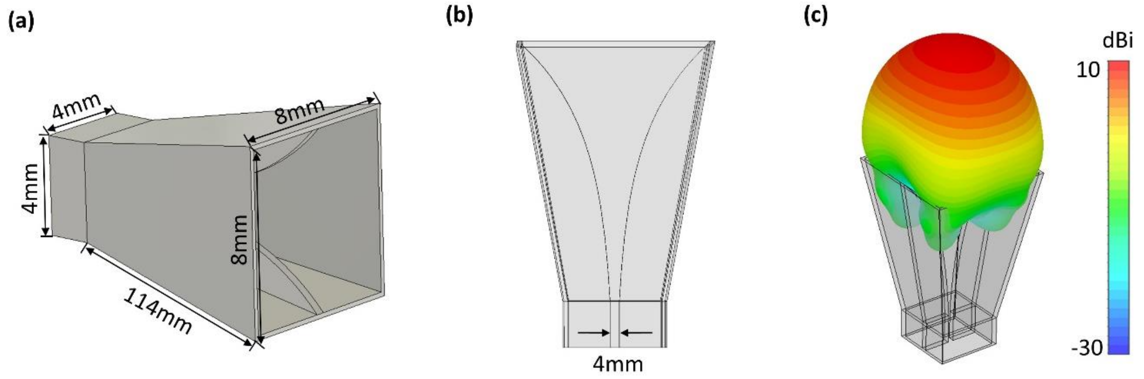

Figure S3. (a) Structure of the broadband double-ridged horn antenna in 3-D view and its geometry parameters. (b) Cross-section view of the antenna. (c) Simulated far-field pattern of the antenna at 3.45GHz.

S4. Description of the Defined Pattern Error

Pattern error is defined as:

$$\boldsymbol{PE}(\boldsymbol{\theta_{an}}) = \frac{\sum_{M=Row_s}^{Row_e}\sum_{N=Columns_s}^{Column_e} K(M,N)\left(RSSP_{user(norm)}^{\theta_{an}}(M,N) - RSSP_{anchor}^{\theta_{an}}(M,N)\right)^2}{\sum_{M=Row_s}^{Row_e}\sum_{N=Columns_s}^{Column_e} K(M,N)}. \quad (1)$$

The calculation region boundary is denoted by Row_s, Row_e, $Column_s$, and $Column_e$ for PE. For simplicity, we limit the DNS estimation to a 1-D scale. Therefore, the specified calculation region consists of central ten rows, with Row_s = 12 and Row_s = 21, respectively. The center of the calculation region is chosen to coincide with the point with the strongest power level in $RSSP_{target}$, as this strongest power point contains more useful DNS information in our strategy. The column that the strongest power point is in is set as $Column_{center}^{RSSP}$. Specifically,

$$\boldsymbol{Column_s} = \begin{cases} Column_{center}^{RSSP} - \frac{Span}{2}, & Column_{center}^{RSSP} - \frac{Span}{2} \geq column_{min} \\ Column_{min}, & Column_{center}^{SP} - \frac{Span}{2} < column_{min} \end{cases} \quad (3)$$

$$\boldsymbol{Column_e} = \begin{cases} Column_{center}^{SP} + \frac{Span}{2}, & Column_{center}^{SP} + \frac{Span}{2} \leq column_{max} \\ Column_{max}, & Column_{center}^{SP} + \frac{Span}{2} > column_{max} \end{cases} \quad (4)$$

where $Span$ is the column span of the calculation region. To prevent the edge effect of the metasurface from unstable DNS estimation, we set the minimum value of $Column_s$ as $Column_{min}$ and the maximum value of $Column_e$ as $Column_{max}$, respectively. Figure 4S gives an example to describe the calculation region intuitively.

As a postprocessing step for accurate DNS estimation, a weighting coefficient $K(M, N)$ based on the linear power level of the target and the anchor is introduced. Specifically, $K(M, N)$ is defined as:

$$\boldsymbol{K(M,N) = 10^{\frac{(RSSP^{\theta an}_{target(norm)}(M,N)+RSSP^{\theta an}_{anchor}(M,N))\times Scale}{10}}} \tag{5}$$

where parameter *Scale* is introduced to tune the correlation degree between the weighting coefficient and the overall power level. Owing to $RSSP_{an}$ being fixed after the implementation of the metasurface, the estimated angle is determined by $Column_{min}$, $Column_{max}$, *Scale,* and *Span*. Thus, we can improve DNS estimation accuracy within a specified area by parameter optimization. The optimization goal is listed below:

Min {Max ($\theta_{deviation}(\theta_{in} = -60°, H = 600$ mm), $\theta_{deviation}(\theta_{in} = -55°, H = 600$ mm)…, $\theta_{deviation}(\theta_{in} = 60°, H = 600$ mm), $\theta_{deviation}(\theta_{in} = -60°, H = 1000$ mm), $\theta_{deviation}(\theta_{in} = -55°, H = 1000$ mm),…, $\theta_{deviation}(\theta_{in} = 60°, H = 1000$ mm))}.

s.t. $0 < Column_{min} < Column^{RSSP}_{center}$, $Column^{RSSP}_{center} < Column_{max} < 32$, $0 < Span < 32$.

where $\theta_{deviation} = |\theta_{estimated} - \theta_{actual}|$. The optimized parameters based on the calculated sensing patterns are listed below: $Column_{min} = 1, Column_{max} = 32, Span_{sp} = 28, Scale = 1.5$. In the experimental verification, the parameters are set as $Column_{min} = 4, Column_{max} = 29, Span = 24, Scale = 2.4$ to match the measured RSSP to improve the DNS estimation accuracy.

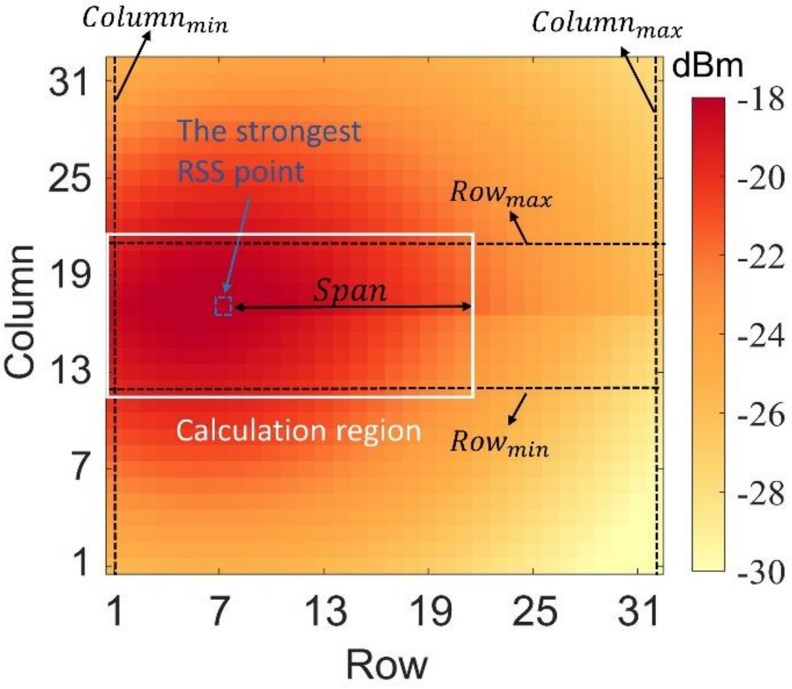

Figure S4. Diagram illustration of the calculation region enclosed by the solid white line under the example of H = 600 mm and θ_{in} = -40°.

S5. *PE* at Other Points within the Specified Area

In Figure S5, *PE* and the corresponding $\theta_{estimated}$ with the target location of (H = 700 mm, $\theta_{in} =$ 17°), (H = 700 mm, $\theta_{in} =$ 33°), (H = 700 mm, $\theta_{in} =$ 52°), (H = 1000 mm, $\theta_{in} =$ 17°), (H = 1000 mm, $\theta_{in} =$ 33°), and (H = 1000 mm, $\theta_{in} =$ 52°) are demonstrated. It can be seen that the $\theta_{estimated}$ is close to the actual angle under different distances H. Although we only optimize the DNS estimation accuracy at the outline of the specified area (H = 600mm and 1000 mm), it is found that the DNS estimation also works well at the locations within the area.

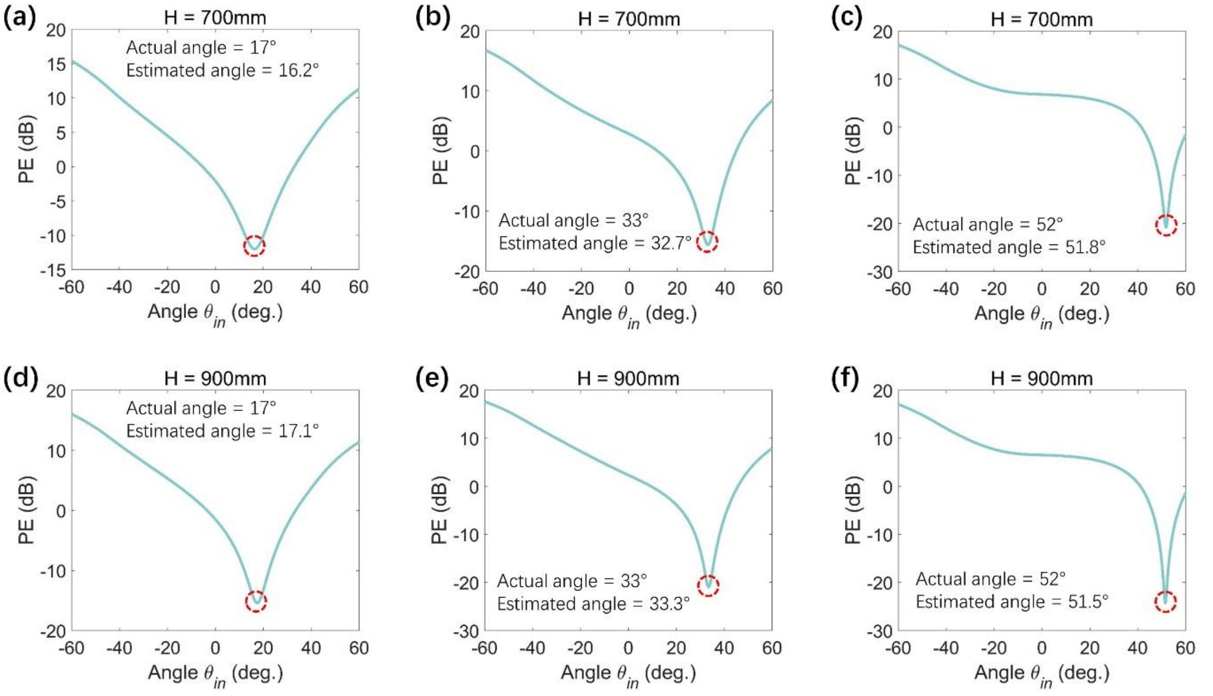

Figure S5. (a)-(f) Pattern error based on the theoretically-calculated RSSP with the target location of (H = 700 mm, $\theta_{in} = 17°$), (H = 700 mm, $\theta_{in} = 33°$), (H = 700 mm, $\theta_{in} = 52$, (H = 900 mm, $\theta_{in} = 17$, (H = 900 mm, $\theta_{in} = 33°$), and (H = 900 mm, $\theta_{in} = 52°$).

S6. Assessment of the Anti-Interference Ability

In real-world indoor scenarios, the EM interference will affect the performance of the system much or less. The interference can be generally divided into two categories: the effects caused by the external power sources and multipath effects generated by the target's own signal.

For analyzing the effects caused by the external power sources, we consider a specific scenario, in which the target power source and an external interference source (both are assumed to be directional point sources without EM scattering) impinge EM waves on the metasurface. As illustrated in Figure S6, the target power source and the external interference source are at the locations of (1000 mm, 0°) and (1200 mm, 45°), respectively. The power level difference between the target source ($Power_t$) and the interference source ($Power_i$) is denoted as $\Delta X = Power_t - Power_i$. The initial phase difference between the target source ($Phase_t$) and the interference source ($Phase_i$) is denoted as $\Delta P = Phase_t - Phase_i$. The first and the second rows in Figure S7 demonstrate the theoretically-calculated RSSPs at the power

level differences $\Delta X = 10$ dBm (low noise), 5 dBm (medium noise), and 2 dBm (high noise) under $\Delta P = 0°$ and 180°, respectively. Although the RSSP suffers significant distortion when ΔX is of 2 dBm (see the third row in Figure S7), the DNS estimation is still good, thus indicating good anti-interference ability.

In order to assess the multipath effects in the indoor environment, we consider three experimental situations, which are having no obstacles around, having an obstacle near the system, and having an obstacle far from the system. Considering the characteristics of real-world obstacles may vary significantly, the human body is chosen to be the obstacle for generality. As illustrated in Figure S8a-c, DNS estimation of the target at (1000 mm, 0°) is taken as an example to assess the multipath effects. The covering area of the human body is depicted by the red shallow. The central positions of the human body in the *x*-*y* coordinate are denoted as red dots. Figure S8b-f shows the measured RSSPs. It is observed that the situations of having no obstacles and having an obstacle far from the system demonstrate similar RSSPs, while the RSSP measured with an obstacle near the system has severe distortion and deformation. As shown in Figure S8e, the phenomenon of diffraction fringe, caused by the superposition of the wave coming from the target and the wave scattered by the obstacle, can be observed. As a result, the DNS estimations for the three situations are 1.2°, 49.5° (wrong), and 3.1°, respectively.

The proposed amplitude-based DNS estimation strategy has performance degradation when the obstacles/scatterers (like the human bodies) are too close to the system. However, we would like to point out that when the target is in the near-field of the metasurface, it is possible to implement an EM environment with minimized interference, allowing our fabricated metasurface to remain applicable in most cases.

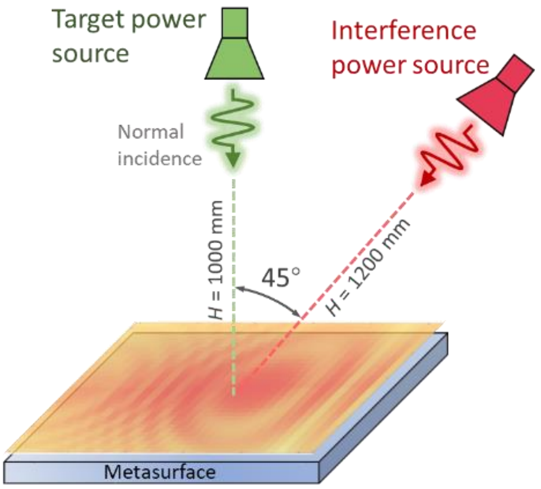

Figure S6. Schematic diagram of the theoretical model to investigate the effects caused by the external interference power source.

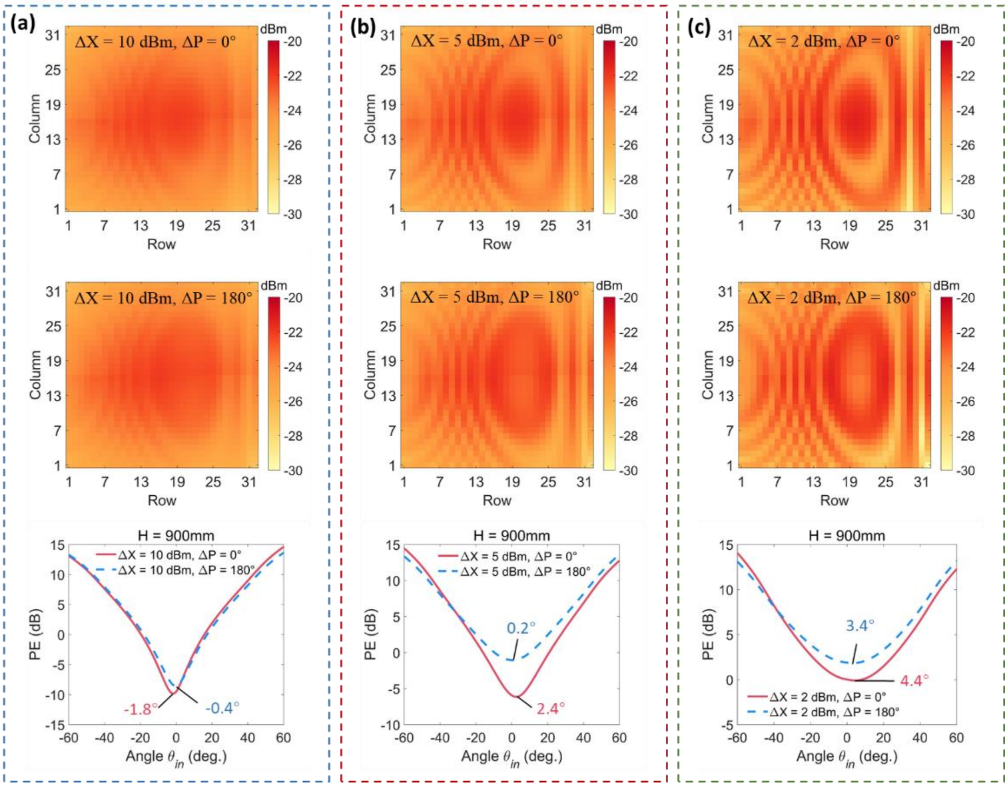

Figure S7. Theorectical-calculated RSSPs and the corresponding estimated angles under different power levels difference between the target and the interference: a) $\Delta X = 10$ dBm, b) $\Delta X = 5$ dBm, and c) $\Delta X = 2$ dBm.

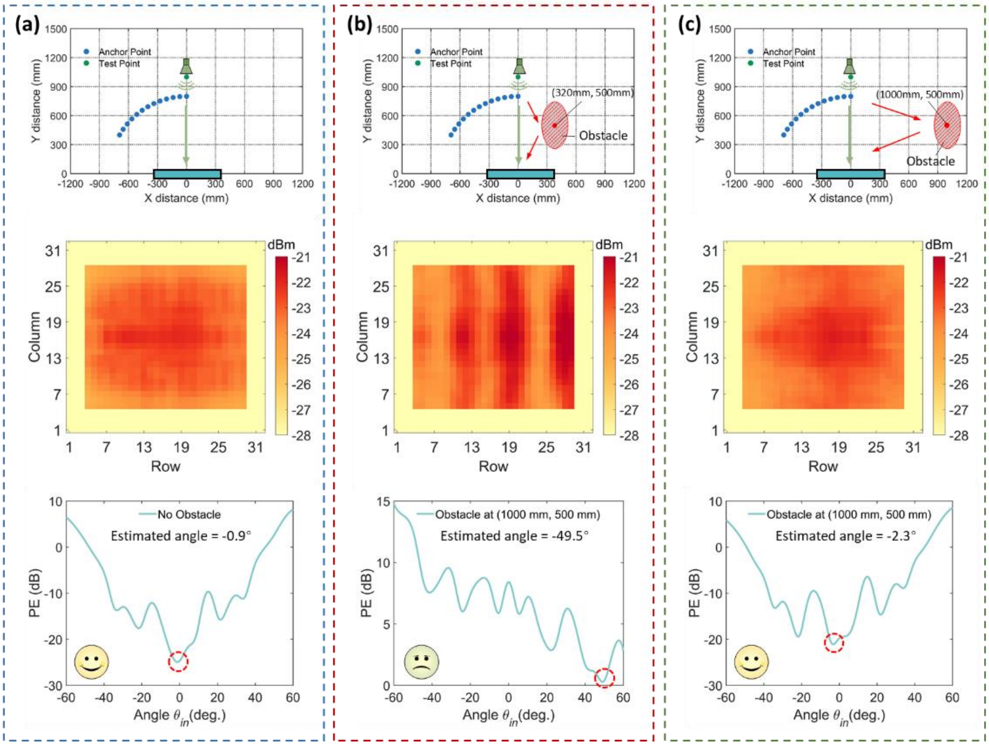

Figure S8. The schematic diagram of the experimental setup, the measured RSSP, and the estimated angle under three situations: a) having no obstacles around, b) having an obstacle near the system, and c) having an obstacle far from the system.

S7. 2-D schematic diagram of the experimental scenario

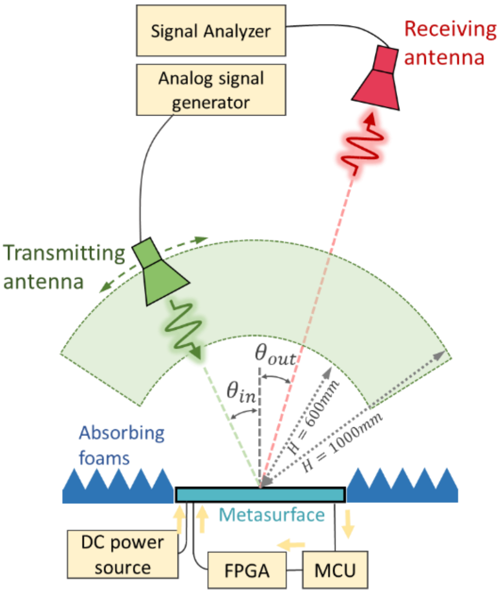

Figure S9. 2-D schematic diagram of the experimental scenario for the adaptive beamforming.
